# Porcine epidemic diarrhoea virus (PEDV) infection activates AMPK and JNK through TAK1 to induce autophagy and enhance virus replication

**DOI:** 10.1080/21505594.2022.2127192

**Published:** 2022-09-27

**Authors:** Jingxiang Wang, Xianjin Kan, Xiaomei Li, Jing Sun, Xiulong Xu

**Affiliations:** a College of Veterinary Medicine, Yangzhou University, Yangzhou, Jiangsu Province, P. R. China; b Institute of Comparative Medicine, Yangzhou University, Yangzhou, Jiangsu Province, P. R. China; c Jiangsu Co-innovation Center for Prevention and Control of Important Animal Infectious Diseases and Zoonosis, Yangzhou University, Yangzhou, Jiangsu Province, China

**Keywords:** Porcine epidemic diarrhoea virus, autophagy, TAK1, AMPK, JNK

## Abstract

Autophagy plays an important role in defending against invading microbes. However, numerous viruses can subvert autophagy to benefit their replication. Porcine epidemic diarrhoea virus (PEDV) is an aetiological agent that causes severe porcine epidemic diarrhoea. How PEDV infection regulates autophagy and its role in PEDV replication are inadequately understood. Herein, we report that PEDV induced complete autophagy in Vero and IPEC-DQ cells, as evidenced by increased LC3 lipidation, p62 degradation, and the formation of autolysosomes. The lysosomal protease inhibitors chloroquine (CQ) or bafilomycin A and Beclin-1 or ATG5 knockdown blocked autophagic flux and inhibited PEDV replication. PEDV infection activated AMP-activated protein kinase (AMPK) and c-Jun terminal kinase (JNK) by activating TGF-beta-activated kinase 1 (TAK1). Compound C (CC), an AMPK inhibitor, and SP600125, a JNK inhibitor, inhibited PEDV-induced autophagy and virus replication. AMPK activation led to increased ULK1^S777^ phosphorylation and activation. Inhibition of ULK1 activity by SBI-0206965 (SBI) and TAK1 activity by 5Z-7-Oxozeaenol (5Z) or by TAK1 siRNA led to the suppression of autophagy and virus replication. Our study provides mechanistic insights into PEDV-induced autophagy and how PEDV infection leads to JNK and AMPK activation.

## Introduction

Porcine epidemic diarrhoea (PED) virus (PEDV) is a single-stranded RNA virus that belongs to the α genera of the *Coronaviridae* family [1]. PEDV primarily infects swine intestinal epithelial cells. Clinical signs include vomiting, watery diarrhoea, and dehydration [2,3]. In the past two decades, PED has spread widely across many Asian countries including China, Thailand, South Korea, and Vietnam and causes heavy economic losses to pig farms [2–4]. These newly isolated PEDV strains are often highly virulent and result in an 80–100% fatality rate in piglets [2,3]. The genome of PEDV is composed of an approximately 28 kb-long RNA [5]. Much of its genome encodes two nonstructural proteins, which are assembled to form an RNA polymerase and direct virus replication and viral gene transcription [5]. About one-third of its remaining genome encodes a non-structural protein (ORF3) and four structural proteins including spike-like protein (S), membrane (M), nucleocapsid (N), and envelope (E) [5]. These proteins are mainly responsible for virus entry into host cells as well as for induction of antiviral immunity [5].

Autophagy is a highly conserved self-degradative cellular process that relies on the protease-enriched lysosomes. Autophagy recycles misfolded “junk” proteins and dysfunctional organelles and plays an important role in defending against intracellular microbes [4,6,7]. Autophagy starts by assembling a crescent-shaped double-membrane fragment, e.g. phagophore, which then extends to wrap the cytosolic cargo and to form a vesicle-like structure designated as the autophagosome [6,7]. Autophagosomes then engage with lysosomes to form autolysosomes where the autophagosome content and the inner autophagosome membrane are degraded by proteases [6,7]. UNC-51-like kinase 1 (ULK1) plays an important role in activating the autophagic pathway. mTOR, a nutrient-sensitive kinase, phosphorylates ULK1 at the serine residue 757 [8,9]. On the contrary, AMPK, an energy-sensitive kinase, phosphorylates ULK1 at several serine residues including S317, S555, and S777 [10,11]. c-Jun terminal kinase (JNK) is a stress-activated protein kinase that phosphorylates Bcl-2 and prevents its binding to Beclin-1, an autophagy protein involved in the formation of the preinitiation complex [12–14]. JNK is activated by a variety of MAP kinase kinase kinases (MAP3Ks) such as MEKK1/2/4, ASK1, and TAK1 [15]. TAK1 is activated by multiple Toll-like receptors through TRAF3 and TRAF6 [16].

Autophagy is a “double-edged sword” in virus replication. It slows down the replication of numerous viruses such as Sindbis virus, picornaviruses, hepatitis C virus (HCV), and human immunodeficiency virus (HIV) by degrading viral proteins, virions or even cellular factors required for virus replication [17]. In contrast, several DNA viruses such as human herpesvirus-1 (HSV-1) and cytomegalovirus (CMV), which can cause persistent infection, have evolved various strategies to evade autophagic destruction [17]. These viruses subvert autophagy by tethering a viral protein with an autophagic component such as Beclin-1 [17]. Numerous RNA viruses such as influenza virus, poliovirus, and flavivirus manipulate autophagosomes to facilitate their replication [18,19]. Coronaviruses are important pathogens that infect human and animal respiratory and gastrointestinal tracts. However, whether autophagy is beneficial or detrimental to the replication of human and porcine coronaviruses such as transmissible gastroenteritis virus (TGEV) or PEDV remains under debate [20–26]. Recent studies suggest that reactive oxygen species (ROS) and two viral proteins, the nonstructural proteins 6 and open reading frame 3 (ORF3) of PEDV, can induce autophagy [21–23,27,28]. The signalling pathway involved in PEDV-induced autophagy are insufficiently understood. Here we present evidence that PEDV infection activates TAK1, which then activates JNK and AMPK to induce autophagy. Inhibition of autophagy suppresses PEDV replication. Our investigation has unveiled a previously unrecognized role of TAK1 in regulating coronavirus-induced autophagy and virus replication.

## Results

### PEDV induces complete autophagy that benefits virus replication

**Figure 1.**
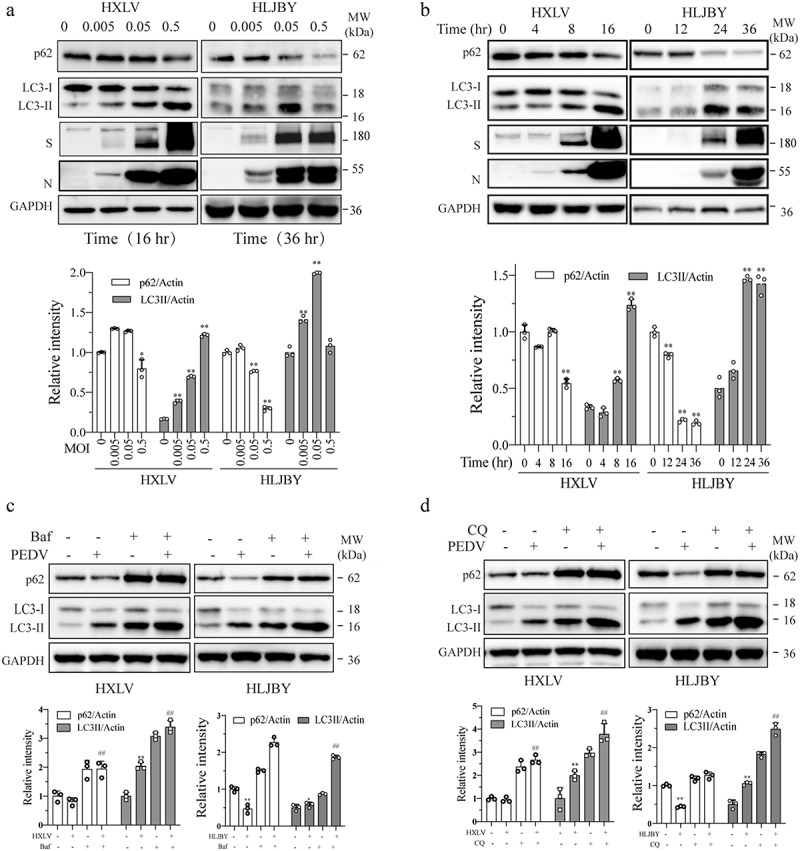
PEDV induces autophagy. Vero and IPEC-DQ cells were infected with the indicated MOI (0, 005, 0.05, and 0.5) of PEDV HXLV and HLJBY strains for 16 hr or 36, respectively (a), or with 0.5 MOI for the indicated time (b). (c & d) Vero and IPEC-DQ cells were mock-infected or infected with PEDV HXLV and HLJBY strains (0.5 MOI each), respectively. After incubation for 8 hr, bafilomycin a (Baf) (20 nM) or chloroquine (CQ) (10 µM) was added and incubated for another 8 hr. LC3 lipidation and the levels of p62, the viral nucleoprotein (N) and spike (S) proteins were analysed by Western blot. β-actin was detected as a loading control. *p < 0.05, **p < 0.01, compared to uninfected controls; ^*#*^*p <* 0.05, ^*##*^*p <* 0.01, compared to PEDV-infected cells.

We first evaluated the ability of PEDV to induce autophagy in Vero and IPEC-DQ cells infected with two different PEDV strains, HXLV and HLJBY, respectively. HXLV is a PEDV isolate with moderate pathogenicity that can readily grow in Vero but not IPEC-DQ cells, a subclone of a porcine IPEC-J2 intestinal epithelial cell line [29,30]. HLJBY, a low pathogenic PEDV strain isolated from a diseased piglet in 2011 [31], can grow in both Vero and IPEC-DQ cells. As shown in Figure 1(a), HXLV and HLJBY viruses dos-dependently increased LC3 lipidation in Vero and IPEC-DQ cells, respectively. Induction of LC3 lipidation by these two viruses were time-dependent (Figure 1(b)). The p62 levels were significantly decreased in HXLV-infected Vero and in HLJBY-infected IPEC-DQ cells in the late cycle of virus infection. The viral S and N proteins were detectable in PEDV-infected cells in a dose- and time-dependent manner. Of note, the N protein of HLJBY virus was detected as a doublet in IPEC-DQ cells, which results from cleavage by caspase-6 or caspase-7 [32].

We next determined if PEDV increased LC3-II levels by inducing functional autophagy or by blocking autophagic flux. We examined the effect of the H-ATPase inhibitor bafilomycin A and the lysosomal protease inhibitor chloroquine (CQ) on LC3-II lipidation and p62 levels in PEDV-infected cells. As shown in Figure 1(c,d) , bafilomycin A (20 nM) and CQ (10 µM) alone significantly increased LC3-II and p62 levels in Vero and IPEC-DQ cells and further increased LC3 lipidation in PEDV-infected cells (Figure 1(d)). p62 levels were slightly decreased in PEDV HXLV strain-infected Vero cells at 16 hour post-infection (hpi) but more remarkably decreased in HLJBY-infected IPEC-DQ cells (Figure 1(c,d)). Bafilomycin A and CQ completely blocked p62 degradation in both cell lines (Figure 1(c,d)).

**Figure 2.**
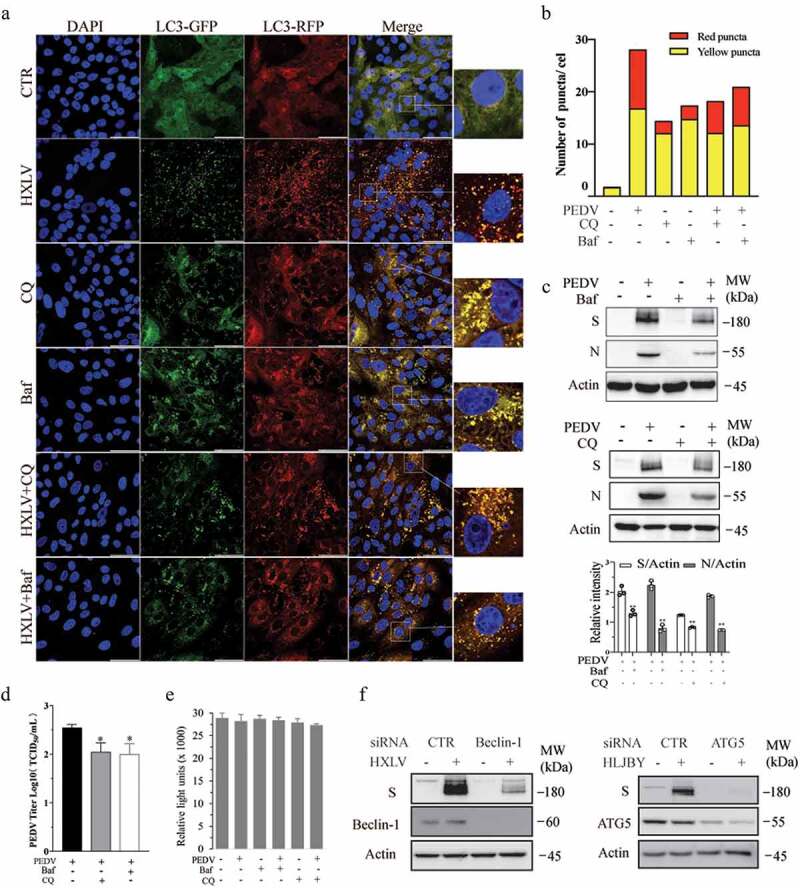
PEDV-Induced autophagy facilitates virus replication. (a) GFP-RFP-LC3-transfected Vero cells were mock-infected or infected with PEDV virus (0.5 MOI). After incubation for 8 hr., CQ (10 µM) and bafilomycin a (20 nM) were added and incubated for another 8 hr. After nuclei staining with DAPI, orange autophagosomal puncta and red autolysosomal puncta were examined and under a confocal microscope and quantified. Scale bar, 50 µm. The means of autolysosomes shown in red bars and autophagosomes shown in orange bars per cell were plotted in a bar graph (b). (c & d) Autophagy facilitates PEDV replication. Vero cells were inoculated with PEDV (0.5 MOI) and then incubated with or without bafilomycin a (20 nM) or CQ (10 µM) for 12 hr. Virus replication was examined by analysing the viral S and N protein levels with Western blot (c). Virus titres in the conditioned media were analysed by measuring the TCID_50_ values (d). The results represent the mean ± SD of four independent experiments. *p < 0.05; **p <0.01 compared to PEDV-infected cells. (e) Effect of bafilomycin a and CQ on Vero cell proliferation. Bafilomycin a (20 nM) and CQ (10 µM) were added into uninfected or PEDV HXLV strain-infected Vero cells and incubated for 12 hr. Cell proliferation was evaluated by using a CellTiter-Glo kit. The mean ± SD of the triplicate from a representative experiment was shown in a bar graph. The experiment was repeated twice with similar results. (f) Vero and IPEC-DQ cells were transfected with Beclin-1 and ATG5 siRNA, respectively. Transfection with a scrambled siRNA was included as a negative control. After incubation for 36 hr, Vero and IPEC-DQ cells were infected with HXLV and HLJBY virus, respectively. Twelve hours later, the levels of the S protein of PEDV and Beclin-1 or ATG5 were detected by Western blot.

Confocal microscopy was conducted to assess the ability of PEDV to induce the formation of autolysosomes. Vero cells were infected with a lentiviral vector encoding an GFP-RFP-LC3 (green fluorescence protein-red fluorescence protein-LC3) reporter. Orange puncta displayed in GFP-RFP-LC3-expressing cells are autophagosomes. Since the GFP signal is quenched in acidic lysosomes, red puncta displayed in GFP-RFP-LC3-expressing cells are autolysosomes, suggesting that cells undergo complete autophagy. As shown in Figure 2(a,b), very few red (0.27 ± 0.08/cell) and orange (1.57 ± 0.12/cell) puncta were seen in uninfected Vero cells. PEDV infection significantly increased the number of orange (15.87 ± 0.94/cell) and red puncta (11.46 ± 0.21/cell), compared to the uninfected control. Bafilomycin A (20 nM) alone dramatically increased the number of orange puncta (14.72 ± 0.8/cell). Bafilomycin significantly decreased the number of red puncta (1.15 ± 0.12%) in PEDV-infected Vero cells. CQ alone (10 µM) increased the number of orange puncta (12.05 ± 1.2/cell). CQ treatment decreased the number of red puncta (3.8 ± 0.26%) in PEDV-infected Vero cells. These observations further suggest that PEDV infection does not block autophagic flux but rather induces functional autophagy.

We next investigated the impact of autophagy on PEDV replication. Bafilomycin (20 nM) significantly reduced the contents of the S and N proteins by 49.6% and 75.2%, respectively, in the cell lysates (Figure 2(c)) and lowered virus titres by 71.2% (Figure 2(d)in the conditioned media of PEDV-infected Vero cells. CQ also reduces the viral S and N protein levels in Vero cell lysates and virus titres in the conditioned media (Figure 2(c,d)). Neither bafilomycin A nor CQ affected the proliferation of uninfected or PEDV-infected Vero cells (Figure 2(e)). The specificity of autophagy inhibition on PEDV replication was verified by knockdown of autophagy-related genes. As shown in Figure 2(f), Beclin-1 and ATG5 siRNA dramatically decreased their expression, compared to that in the cells transfected with scrambled siRNA. Inhibition of Beclin-1 and ATG5 significantly decreased the levels of viral S proteins (Figure 2(f)). These observations collectively suggest that autophagy benefits PEDV replication.

### JNK activation contributes to PEDV-induced autophagy and enhances virus growth

**Figure 3.**
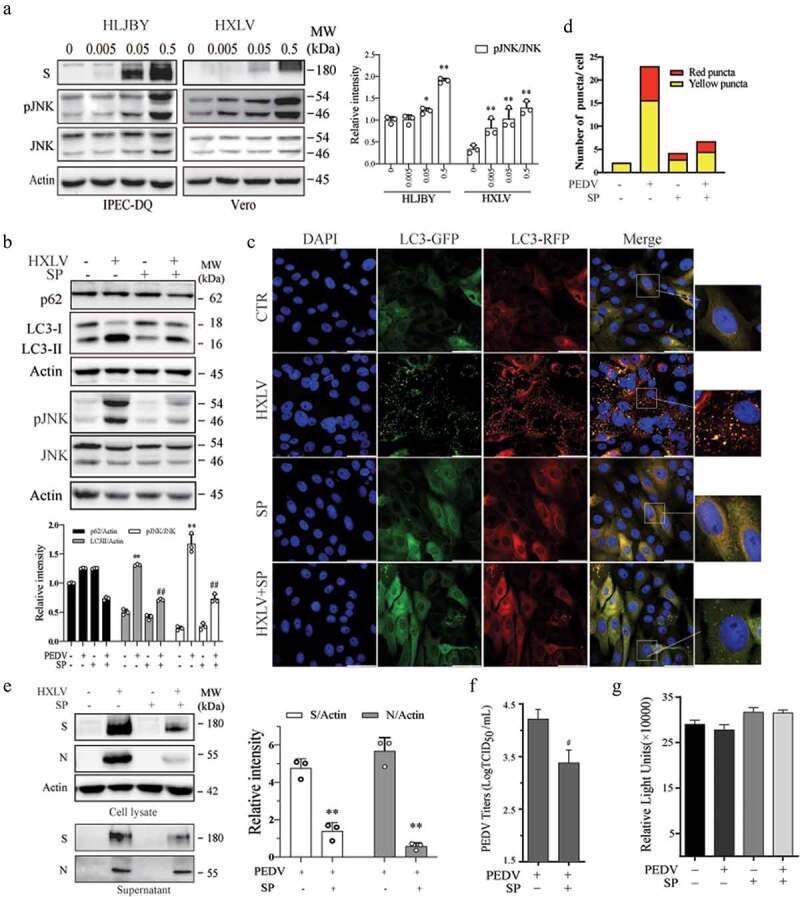
JNK activation contributes to PEDV-induced autophagy. (a) IPEC-DQ and Vero cells were infected with PEDV HLJBY and HXLV strains and incubated for 24 and 12 hr, respectively. JNK phosphorylation and total JNK protein levels were analysed by Western blot. (b) Vero cells inoculated with 0.5 MOI PEDV were incubated without or with SP600125 (SP) (10 µM) for 12 hr. The levels of LC3, p62, and JNK phosphorylation were analysed by Western blot. *p <0.05, compared to uninfected controls; ^*#*^*p <* 0.05, ^*##*^*p <0.01*, compared to PEDV-infected cells. (c & d) GFP-RFP-LC3-transfected Vero cells were mock-infected or infected with PEDV HXLV virus (0.5 MOI) and incubated for 8 hr. SP600125 (10 µM) was then added and incubated for another 8 hr. After nuclei staining with DAPI, orange autophagosomal puncta and red autolysosomal puncta were examined and quantified under a confocal microscope (c). The mean numbers of autolysosomes shown in red bars and autophagosomes shown in orange bars per cell were plotted in a bar graph (d). (e & f)Vero cells infected with PEDV (0.5 MOI) were treated with dimethyl sulphoxide (DMSO 0.5%) or SP600125 (10 µM) for 12 hr. The S and N proteins in the cell lysates and conditioned media were analysed by Western blot (e). The levels of the S and N proteins relative to β-actin was analysed and plotted in a bar graph. The conditioned media of PEDV-infected cells were titrated for the TCID_50_ values (f). ^*#*^*p <* 0.05, ^*##*^*p <* 0.01, compared to PEDV-infected cells. (g) SP600125 does not affect cell Viability. Uninfected and PEDV-infected Vero cells were treated with DMSO or SP600125 (10 µM) for 12 hr. Cell viability was analysed by using a CellTiter-Glo kit. The mean ± SD of the triplicate from a representative experiment was shown in a bar graph. The experiment was repeated twice with similar results.

Accumulating evidence suggests that JNK activation contributes to viral infection-induced autophagy [33,34]. We first examined the ability of PEDV to activate JNK in Vero and IPEC-DQ cells. As shown in Figure 3(a), JNK phosphorylation was significantly increased in a dose-dependent manner in IPEC-DQ cells infected with the HLJBY strain and in Vero cells infected with the HXLV strain. SP600125 (10 µM), a specific inhibitor of JNK, did not significantly affect LC3 lipidation in uninfected Vero cells but largely blocked PEDV-induced LC3 lipidation (Figure 3(b)). SP600125 inhibited PEDV-induced JNK phosphorylation at 12 hpi (Figure 3(b)). Consistently, SP600125 blocked PEDV-induced autolysosome and autophagosome formation as there were fewer orange and red puncta in PEDV-infected Vero cells in SP600125-treated cells, compared to the untreated control (Figure 3(c,d)).

We next determined if JNK activation was involved in PEDV growth. As shown in Figure 3(e), SP600125 treatment decreased the S and N protein levels in the cell lysates by 59.8% and 50.8%, respectively. SP600125 also significantly decreased the viral protein levels (Figure 3(e)) and virus titres (Figure 3(f)) in the culture media of PEDV-infected Vero cells by 85.4%. SP600125 did not affect Vero cell proliferation (Figure 3(g)). To exclude the possibility that inhibition of PEDV-induced autophagy by SP600125 was caused by inhibition of virus replication, SP600125 was added 6 hr after PEDV infection and then incubated for another 6 hr. Under this experimental setting, SP600126 was no longer able to inhibit HXLV replication but was still able to block JNK phosphorylation and LC3 lipidation (Supplementary Figure S1a).

### AMPK activation contributes to PEDV-induced autophagy and enhances virus replication

**Figure 4.**
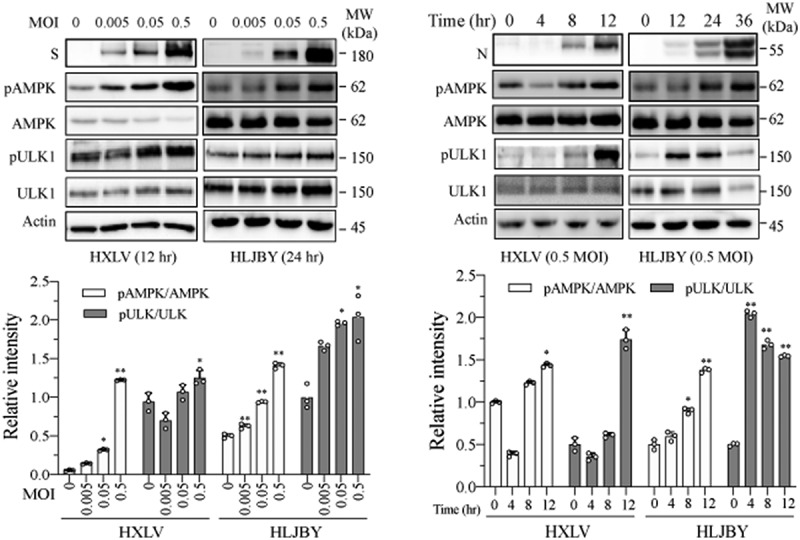
PEDV infection increases the levels of AMPK and ULK1 phosphorylation. Vero and IPEC-DQ cells were mock-infected or infected with HXLV and HLJBY viruses for 12 or 24 hr, respectively, or with 0.5 MOI of HXLV virus for the indicated lengths of time. AMPK^T172^ and ULK1^S777^ phosphorylation was detected by Western blot. *p <0.05, **p <0.01, compared to uninfected controls.

**Figure 5.**
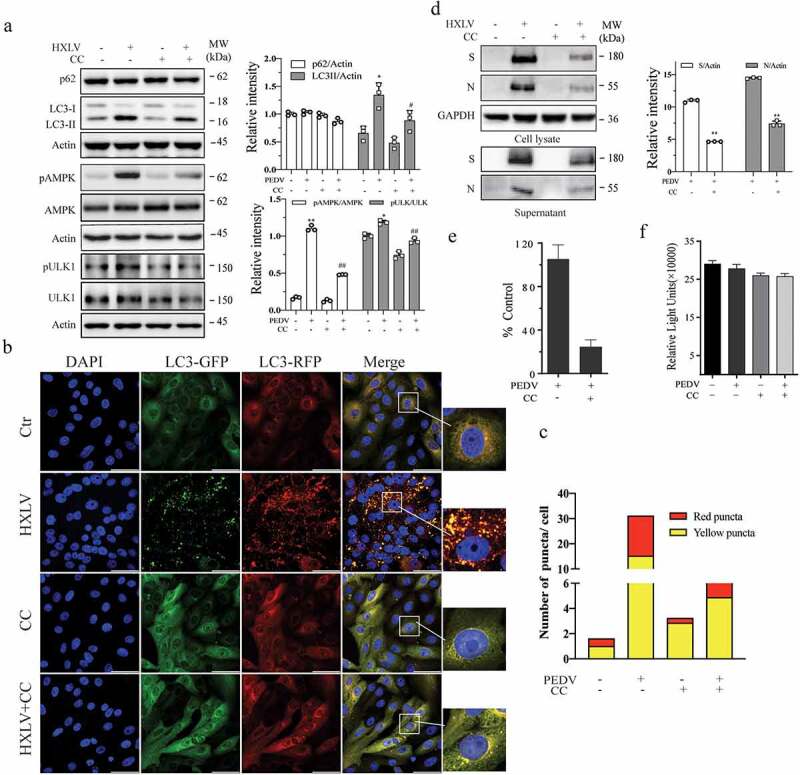
AMPK inhibition suppresses autophagy and PEDV replication. (a) Compound C (CC) suppresses autophagy in PEDV-infected cells. Mock- or HXLV virus-infected Vero cells were treated with DMSO (0.5%) or CC (2 µM) for 12 hr. LC3 lipidation, p62, and AMPK^T172^ and ULK1^S777^ phosphorylation were detected by Western blot. (b & c) CC suppresses autophagosome formation in PEDV-infected cells. GFP-RFP-LC3-transfected Vero cells were mock-infected or infected with HXLV virus (0.5 MOI). After incubation for 8 hr, the cells were treated with DMSO (0.5%) or CC (2 µM) for 8 hr. After nuclei staining with DAPI, orange autophagosomal puncta and red autolysosomal puncta were examined under a confocal microscope and quantified (b). The mean numbers of autolysosomes shown in red bars and autophagosomes shown in orange bars per cell were plotted in a bar graph (c). (d & e) CC inhibits virus growth. Mock or PEDV-infected Vero cells were treated with DMSO (0.5%) or CC (2 µM) for 12 hr. Viral proteins in the conditioned media and in cell lysates were analysed by Western blot (d). The virus titres in the conditioned media were analysed for the TCID_50_ values (e). *p < 0.05, **p < 0.01. (f) CC does not affect Vero cell proliferation. Uninfected and PEDV-infected Vero cells were incubated with DMSO (0.5%) or CC (2 µM) for 12 hr and then examined for cell viability by using a CellTiter-Glo kit. The data represents the mean ± SD of the triplicate from a representative experiment, which was repeated twice with similar results.

AMPK activation triggers autophagy by phosphorylating ULK1 at multiple sites including S317, S555, and S777 [35]. We investigated if AMPK was activated in PEDV-infected Vero cells. As shown in Figure 4, AMPK and ULK1^S777^ phosphorylation was increased in a time- and dose-dependent manner in HXLV virus-infected Vero cells and in HLJYB virus-infected IPEC-DQ cells. PEDV infection had no effect on ULK1^S555^ phosphorylation in Vero cells (data not shown). We next evaluated the impact of AMPK activation on PEDV-induced autophagy and virus growth. The AMPK-specific inhibitor compound C (CC) (2 µM) blocked PEDV virus-induced AMPK and ULK1^S777^ phosphorylation and LC3-II levels (Figure 5(a)). CC also prevented PEDV-induced autophagosome and autolysosome formation, as indicated by the presence of fewer orange and red puncta (Figure 5(b,c)). CC decreased the S and N protein levels in the cell lysates by 57% and 51.4%, respectively, and significantly decreased the S and N protein levels in the culture media of PEDV-infected Vero cells (Figure 5(d)). Consistently, CC also lowered the titre of PEDV in the culture media of Vero cells by 76.9% (Figure 5(e)). CC had no effect on Vero cell proliferation (Figure 5(f)). To determine if inhibition of PEDV-induced autophagy by CC was due to inhibition of virus replication, CC was added 6 hr after PEDV infection and then incubated for another 6 hr. Under this experimental setting, CC was no longer able to inhibit HXLV replication but was still able to block AMPK phosphorylation and LC3 lipidation (Supplementary Figure S1b).

### ULK1 activation contributes PEDV-induced autophagy and virus growth

**Figure 6.**
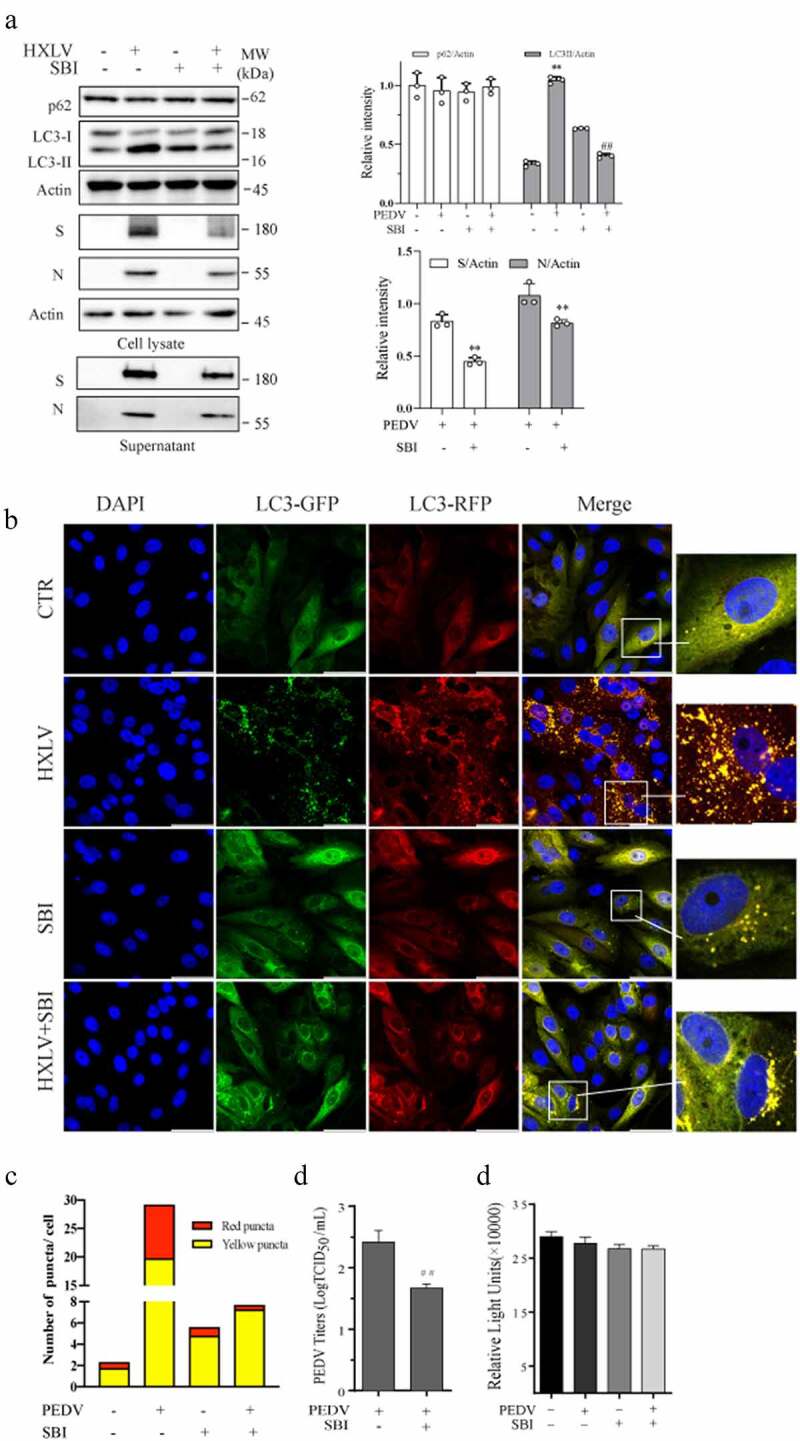
ULK1 activation contributes to PEDV-induced formation of autolysosomes and virus replication. (a) SBI -0,206,965 (SBI) inhibits PEDV-induced autophagy. Uninfected or PEDV-infected Vero cells were treated with DMSO (0.5%) or SBI (1 µM) for 12 hr. LC3 and p62 were detected by Western blot. (b & c) GFP-RFP-LC3-transfected Vero cells were mock-infected or infected with PEDV (0.5 MOI) and incubated for 8 hr. The cells were then treated with DMSO (0.5%) or SBI (1 µM) for another 8 hr. After nuclei staining with DAPI, orange autophagosomal puncta and red autolysosomal puncta were examined under a confocal microscope and quantified (b). The numbers of puncta were calculated and presented as a bar graph (c). (d)uninfected or PEDV-infected Vero cells were treated with DMSO (0.5%) or SBI (1 µM) for 12 hr. Viruses released into the conditioned media were titrated by measuring TCID_50_ values. Data are the mean ± SD of three independent experiments. (e) SBI does not affect cell viability. Uninfected or PEDV-infected Vero cells were treated with DMSO (0.5%) or SBI (1 µM). After incubation for 12 hr, cell viability was examined by using a CellTiter-Glo kit. Data represent the mean ± SD of the triplicate from a representative experiment, which was repeated twice with similar results.

Having shown that PEDV increased ULK1^S777^ phosphorylation, we investigated the effect of ULK1 activation on PEDV-induced autophagy. As shown in Figure 6(a), SBI-0206965 (SBI) (1 µM), a ULK1-specific inhibitor, increased LC3-II levels. SBI decreased the formation of autophagosomes and autolysosomes in PEDV-infected cells (Figure 6(b,c)). SBI decreased the S and N protein levels in cell lysates by 43.4% and 36.1%, respectively, and in the conditioned media (Figure 6(a)), and decreased the TCID_50_ values in the media of PEDV-infected Vero cells by 82.2% (Figure 6(d)). SBI did not affect Vero cell proliferation (Figure 6(e)).

### TAK1 activation contributes to PEDV-induced autophagy and virus growth

**Figure 7.**
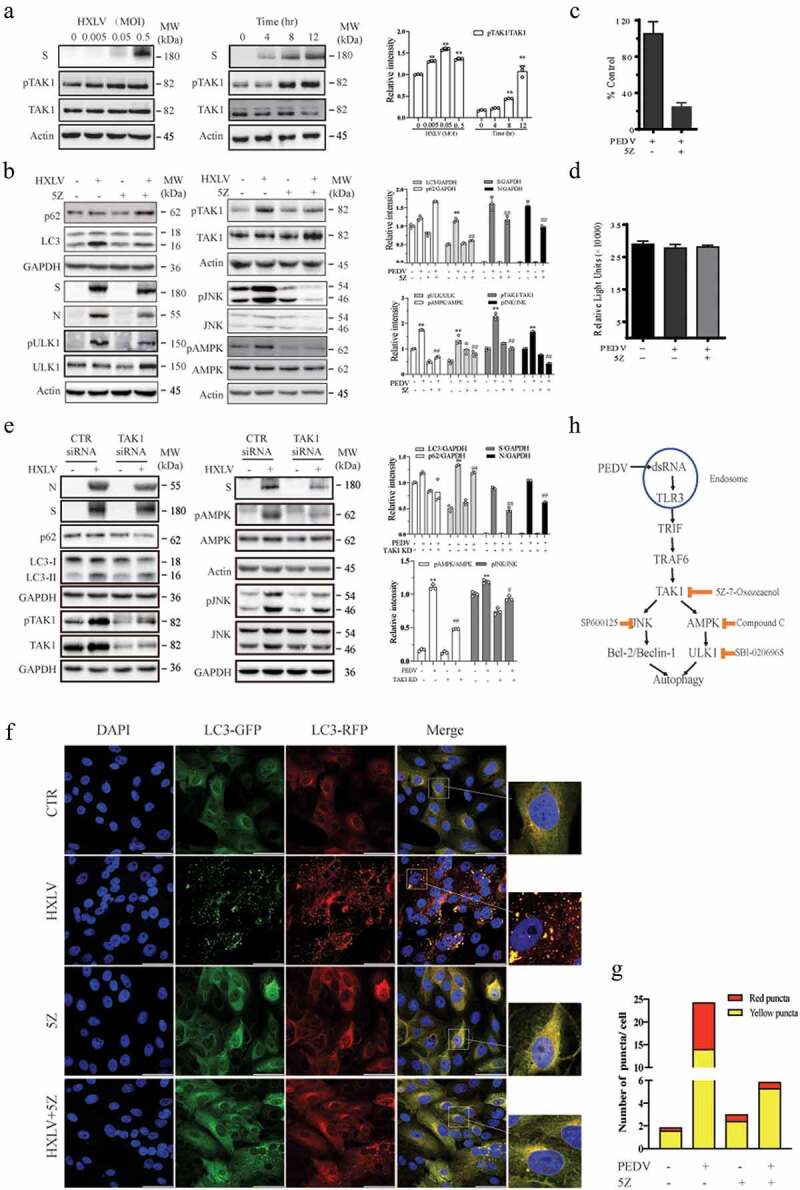
TAK1 activation contributes to PEDV-induced formation of autolysosomes and virus replication. (a) Vero cells were infected with the indicated MOI (0, 005, 0.01, 0.5) of PEDV HXLV strain for 12 hr or with 0.5 MOI for the indicated length of time. TAK1^S412^ phosphorylation, total TAK1 protein, and β-actin were detected by Western blot. (b) Uninfected or PEDV-infected Vero cells were treated with DMSO (0.5%) or 5Z (2 µM) and incubated for 12 hr. TAK1^S412^, AMPK, and JNK phosphorylation, LC3 lipidation, p62, and viral protein levels were analysed by Western blot. Viruses released into the conditioned media were titrated by measuring the TCID_50_ values (c). (d) 5Z has no effect on Vero cell viability. Uninfected or PEDV-infected Vero cells were treated with DMSO (0.5%)or 5Z (2 µM). After incubation for 12 hr, cell viability was quantified by using a CellTiter-Glo kit. (e) TAK knockdown blocks PEDV-induced autophagy. Vero cells were transfected with control or TAK1 siRNA were then incubated for 36 hr. After infection with HXLV virus (0.5 MOI) for 12 hr. LC3, p62, viral N and S proteins, TAK1^S412^ and other phosphorylated proteins were detected with the indicated antibodies. *p < 0.05, **p < 0.01, compared to uninfected control; ^#^*p* <0.05,^##^*p* <0.01, compared to PEDV virus-infected cells. (f & g) GFP-RFP-LC3-transfected Vero cells were mock-infected or infected with PEDV (0.5 MOI). After incubation for 8 hr, the cells were treated with DMSO (0.5%) or 5Z (2 µM) d and incubated for another 8 hr. After nuclei staining with DAPI, orange autophagosomal puncta and red autolysosomal puncta were examined under a confocal microscope and quantified (f). The mean numbers of autolysosomes shown in red bars and autophagosomes shown in orange bars per cell were plotted in a bar graph (g). (h) the schematic model of PEDV-induced autophagy. PEDV viral RNA binds to endosomal TLR3, which then engages with TRIF and activates TRAF6 and its downstream kinase TAK1. TAK1 activates AMPK and JNK activation. AMPK phosphorylates ULK1 at S777 and activates it. JNK phosphorylates Bcl-2, leading to its dissociation with Beclin-1. Free Beclin-1 is then assembled into the pre-initiation complex. ULK1 phosphorylates VPS34, a class III PI-3 kinase in the pre-initiation complex, to induce autophagy. In addition to the TAK1-AMPK/JNK axis, accumulating evidence suggests that other signalling pathways such as ER stress and the PI-3 kinase pathway may participate in mediating PEDV-induced autophagy.

TAK1 plays an important role in activating JNK and AMPK to induce autophagy [16,36–38]. Here we assessed the status of TAK1 activation in PEDV-infected Vero cells and its impact on PEDV-induced autophagy. As shown in Figure 7(a), PEDV induced TAK1^S412^ phosphorylation in a dose- and time-dependent manner. 5Z-7-Oxozeaenol (5Z), a TAK1- specific inhibitor, blocked PEDV virus-induced TAK1, JNK, AMPK, and ULK1 phosphorylation and LC3 lipidation (Figure 7(b)). 5Z decreased S and N protein levels in the cytoplasmic fraction of PEDV-infected Vero cells by approximately 30.9% and 40.5% (Figure 7(b)) and lowered the titre of PEDV in the conditioned media of Vero cells by 76.9% (Figure 7(c)). 5Z did not affect Vero cell viability (Figure 7(d)). To rule out the possibility that 5Z blocked PEDV-induced autophagy by suppressing virus replication, we postponed the addition of 5Z (5 µM) in HXLV virus-infected Vero cells by 6 hr after PEDV infection. Under this experimental setting, 5Z was no longer able to inhibit HXLV replication but was still able to block TAK1 and ULK1 phosphorylation and LC3 lipidation (Supplementary Figure S1c). This suggests that inhibition of TAK1 suppresses autophagy, leading to the suppression of PEDV replication.

To ascertain the role of TAK1 in mediating PEDV-induced autophagy, we tested if TAK1 silencing also repress PEDV-induced autophagy. We found that TAK1 siRNA down-regulated TAK1 expression effectively in uninfected or PEDV-infected Vero cells (Figure 7(e)). TAK1 knockdown blocked PEDV-induced LC3 lipidation and the phosphorylation of JNK and AMPK (Figure 7(e)). 5Z also blocked PEDV- induced autophagosome and autolysosome formation, as indicated by significantly lower numbers of the orange and red puncta (Figure 7(f&g)). These observations collectively suggest that TAK1 plays an important role in activating AMPK and JNK and contributes to PEDV-induced autophagy.

## Discussion

Autophagy is an integral part of host defence against invading intracellular microbes. However, viruses have evolved diverse strategies to avoid autophagic destruction and sometimes can even manipulate the autophagic pathway to facilitate virus growth [39]. Many coronaviruses in the *Coronaviridae* family such as PEDV, TGEV, the acute respiratory syndrome (SARS) virus, SARS-CoV-2, and the Middle East respiratory syndrome (MERS) virus are important pathogens that cause severe respiratory and gastrointestinal infections in humans and animals [40,41]. Our present study aims at understanding the mechanisms of PEDV-induced autophagy and its impact on virus replication. We provide unequivocal evidence that PEDV induced degradative autophagy in Vero and IPEC- DQ cells; Activation of JNK and AMPK contributed to PEDV-induced autophagy and virus replication; Inhibition of autophagy suppressed PEDV replication. We further demonstrate that TAK1 primed JNK and AMPK activation and enhanced virus replication. Our observations collectively suggest that the TAK1-AMPK/JNK axis plays a central role in PEDV-induced autophagy.

Although PEDV-induced autophagy has been studied by several independent investigators, whether PEDV induces functional autophagy remains controversial. Guo et al. [23] demonstrated earlier that a virulent variant strain of PEDV isolated from a suckling piglet with acute diarrhoea induces complete autophagy in Vero cells, as evidenced by increased LC3 lipidation, slightly decreased p62 levels in the late life cycle of virus infection, and the presence of double-membranous vesicles. However, most autophagosomes are not engaged with lysosomes since a great number of puncta in PEDV-infected Vero cells expressing the GFP-RFP-LC3 gene display orange fluorescence (autophagosomes) [23]. It is not clear if bafilomycin and CQ blocks PEDV-induced p62 degradation and increases PEDV-induced LC3 lipidation since a critical control of bafilomycin or CQ treatment alone is lacking in this study [23]. Sun et al. [27] reported that a PEDV variant isolated by these investigators induces LC3 lipidation as well as p62 degradation at 18 hpi or later. In contrast, a recent study by Park et al. [26] showed that a PEDV field isolate induces LC3 lipidation and slightly increased p62 levels in Vero cells. These authors suggest that PEDV induces autophagosome formation but inhibits autolysosome formation, even though a small fraction of red puncta can be seen in PEDV-infected Vero cells [26]. Our present study shows that the HXLV strain of PEDV did not significantly decrease p62 levels in Vero cells in the early hours of infection but did slightly decrease p62 levels at 16 hpi. The HLJBY strain decreased p62 levels in IPEC-DQ cells more effectively than the HXLV strain in Vero cells (Figure 1(a,b)). Confocal microscopic analysis revealed that, while a large proportion of puncta displayed orange fluorescence, more than one third puncta were red fluorescent, suggesting the formation of autolysosomes. These observations suggest that PEDV may induce the fusion of a fraction of autophagosomes with lysosomes and leave the majority of autophagosomes unfused with lysosomes. It should be noted that the efficiency of PEDV in inducing complete autophagy could also be cell type- and virus strain-dependent.

p62, an autophagic receptor that binds LC3-II, is degraded by lysosomal proteases in autolysosomes [42]. p62 downregulation is often considered a hallmark of complete autophagy [43]. However, numerous studies have shown that JNK activation upregulates p62 transcription, leading to increased p62 levels in cells undergoing functional autophagy. For example, dehydroepiandrosterone and resveratrol induce complete autophagy even though p62 levels are increased [43–45]. We recently reported that p62 levels are increased in anticancer drug-induced autophagy [46]. In addition, TAK1 can prevent autophagy-mediated p62 degradation by inhibiting p62 translocation into autophagosomes [47]. Therefore, p62 should not be viewed as the sole indicator of functional autophagy.

Although several members of the *Coronaviridae* family can induce autophagy, its effects on virus replication are inconsistent [17,20,48]. For example, infectious bronchitis virus (IBV) induces autophagy in Vero cells but does not affect virus replication [49]. TGEV induces complete and functional autophagy in TS and PK15 cells [50]. Inhibition of autophagy leads to increased TGEV replication, suggesting that autophagy negatively regulates virus replication [50]. In the present study, we showed that inhibition of autophagy by bafilomycin or CQ or by Beclin-1 and ATG5 knockdown suppressed PEDV replication. Consistent with our observations, several prior studies showed that autophagy benefits PEDV replication [21,23,27,28]. In contrast, Ko et al. [24] reported that induction of autophagy by rapamycin restricts PEDV replication. It is not clear if the inhibitory effect of rapamycin on PEDV replication is due to inhibition of viral protein synthesis by inhibiting 4E-BP and S6 phosphorylation.

It is well documented that JNK activation is involved in the initiation of autophagy. In nutrient-depleted cells, JNK1 phosphorylates Bcl-2 at T69, S70, and S87 [13,14]. Phosphorylated Bcl-2 dissociates with Beclin 1 and allows it to participate in the initiation of autophagy [13,14,51,52]. Several viruses such as influenza virus, adenovirus, and Sendai virus also activate JNK and phosphorylate Bcl-2 to facilitate the induction of autophagy [51–54]. JNK is also required for autophagy induced by the X protein of hepatitis B virus [34] and cytosine-phosphate-guanine (CpG) [55]. JNK activation can facilitate the replication of a variety of viruses. For example, activation of JNK is required for efficient H5N1 influenza A virus replication [51]. ERK, p38, and JNK activation supports PEDV replication in Vero cells [56,57]. Consistent with these observations, our present study shows that JNK was activated in PEDV-infected IPEC-DQ and Vero cells, and that SP600125, a JNK-specific inhibitor, significantly inhibited autophagy and PEDV replication. Since autophagy is involved in stimulating PEDV replication, we speculate that JNK activation aids PEDV replication in part by stimulating autophagy.

Accumulating evidence suggests that some viruses can activate AMPK to mediate virus-induced autophagy. For example, the type 2 porcine circovirus activates AMPK and subsequently inhibits mTOR activity, leading to the induction of autophagy in PK-15 cells [58]. Dengue virus induces autophagy by activating AMPK in HepG2 cells [59]. Respiratory syncytial virus activates AMPK, induces ULK1 S317 and S555 phosphorylation, and subverts mTOR activity [60]. The HN and F proteins of Newcastle disease virus induce the formation of syncytia and autophagy by activating AMPK [61]. The nonstructural protein p17 of avian reovirus activates AMPK and subsequently decreases ULK1^S757^ phosphorylation, leading to autophagy induction [62]. How AMPK is activated by these viruses is not known. Our present study shows that AMPK was activated in PEDV-infected Vero cells. AMPK activation led to increased ULK1^S777^ phosphorylation. Further study revealed that inhibition of AMPK activity by CC and inhibition of ULK1 activity by SBI decreased PEDV replication. These observations suggest that AMPK activation plays an important role in mediating PEDV-induced autophagy and supports virus replication.

TAK1 has been increasingly recognized as a critical kinase capable of activating AMPK and inducing autophagy in various settings [16]. For instance, TAK1 activation plays an important role in inducing autophagy in tumour necrosis factor-related apoptosis-inducing ligand (TRAIL)-stimulated human epithelial cells [63], in TGF-beta-stimulated murine mesangial cells [64], in VEGF-stimulated endothelial cells [65], and in H5N1 influenza A virus-infected 293T cells [52]. TAK1 is also responsible for S6K1 inhibition-induced and Salmonella-induced AMPK activation and autophagy [36,37]. Wang et al. recently reported that two enteroviruses, CVA16 and EV71, induce TAK1^T187^ and TAK1^S192^ phosphorylation and activate it through TLR3 [66]. Our present study shows that PEDV infection induced TAK1^S412^ phosphorylation and activation, most likely through TLR by viral RNA. We further show that TAK1 was responsible for PEDV-induced JNK and AMPK activation. Using a TAK1-specific inhibitor and TAK1 siRNA, we provide evidence that inhibition of TAK1 activity blocked PEDV-induced AMPK and JNK activation, autophagy, and virus replication (Figure 7). While our findings collectively suggest that TAK1 activation plays a critical role in PEDV-induced autophagy, it should be noted that the mechanisms by which PEDV induces autophagy could be complex. For example, ER stress induced by ROS and the ORF3 of PEDV is required for PEDV-induced autophagy [22], inhibition of the PI-3 kinase pathway by the ORF6 of PEDV may also promote autophagy [21]. Our investigation suggests that autophagy induced by PEDV involves the activation of AMPK and JNK by TAK1 (Figure 7(h)). Our study unveils a previously unrecognized pathway that mediates PEDV-induced autophagy.

## Materials and methods

### Reagents

5Z-7-Oxozeaenol (5Z) was obtained from Tocris Bio-Techne (Shanghai, China). Anti-ULK1^S777^ (ABC213), SBI -0,206,965, bafilomycin, and chloroquine (CQ) were purchased from Sigma-Aldrich (Shanghai, China). Compound C was purchased from Selleck Chemicals LLC (Houston, TX, USA). β-actin and GAPDH (glyceraldehyde 3-phosphate dehydrogenase) antibodies were purchased from Santa Cruz Biotechnology, Inc. (Santa Cruz, CA, USA). SP600125 and antibodies for ULK1 (8054S), ULK1^S555^ (5869S), AMPK (5831S), AMPK^T172^ (2535S), TAK1 (5206S), TAK1^S412^ (9339S), JNK (9252S), pJNK (4668S), Beclin-1 (3495S), LC3 (3868S), and p62 (5114S) were purchased from Cell Signalling Technology (Danvers, MA, USA). Monoclonal antibodies for PEDV S and N proteins were a courteous gift of Dr. Ying Fang, College of Veterinary Medicine, University of Illinois at Urbana-Champaign, Urbana, Illinois, USA.

### Cell culture and viruses

Vero cells (CCL-81), an African green monkey kidney cell line, were obtained from the American Tissue Culture Collection (Manassas, VA). This cell line was cultured in complete αMEM medium supplemented with streptomycin and penicillin (100 U/ml), sodium pyruvate (1 mM), L-glutamine (2 mM), foetal bovine serum (FBS) (10%). IPEC-DQ cells, a subclone of the IPEC-J2 porcine intestinal epithelial cell line, were a courteous gift of Dr. Dongwan Yoo, College of Veterinary Medicine, the University of Illinois at Urbana-Champaign, Urbana, Illinois, USA. This cell line was cultured in RPMI 1640 media containing 10% FBS as previously reported [29]. The HXLV strain is a pathogenic PEDV isolate obtained from Liyuan Biotechnology Inc., Nanning, Guanxi Province, China. HLJBY, a low pathogenic PEDV strain isolated from a diseased piglet in 2011 [31], was kindly provided by Dr. Changchao Huan, College of Veterinary Medicine, Yangzhou University, China. During and after PEDV inoculation, TPCK trypsin (8 µg/ml) were added into the culture media of Vero cells [31]. HLJBY but not HXLV virus can be propagated in IPEC-DQ cells [30]. After infection with PEDV (0.5 MOI), Vero and IPEC-DQ cells were cultured in the absence or presence of various inhibitors as indicated in figure legends. PEDV released into the conditioned media was titrated by infecting Vero cells with a 10-fold serial dilution (10^1^ to 10^8^). Virus titres based on the 50% tissue culture infection dose (TCID_50_/0.1 ml) were calculated with the Reed and Muench method. The results in different treatment groups were statistically analysed based on the mean ± standard deviation (SD) of four independent experiments.

### Autophagosome analysis

Vero cells infected with a lentiviral vector encoding GFP-RFP-LC3 were screened in the media containing puromycin. GFP-RFP-LC3-positive cells were mock infected or infected with PEDV virus (0.5 MOI). After incubation for 8 hr, various inhibitors including bafilomycin A (20 nM), chloroquine (10 µM), SP600125 (10 µM), compound C (CC) (2 µM), SBI (1 µM), and 5Z (2 µM) were added and incubated for another 8 hr. The formation and quantification of autophagosomes and autolysosomes were analysed as previously described [51].

### Gene knockdown

Scrambled control siRNA was obtained from Life Technologies (Invitrogen Life Technologies, Grand Island, NY, USA). TAK1 (#6317S), Beclin-1 (#6222S), and ATG5 (6345S) siRNA were purchased from Cell Signalling Technology (Danvers, MA, USA). Transfection of Vero cells with siRNA was carried out with Lipofectamine RNAiMAX transfection reagent (Invitrogen Life Technologies, Grand Island, NY, USA) following the manufacturer’s instruction. Thirty-six hours later, cells were mock infected or infected with PEDV (0.5 MOI) and then incubated for 12 hr. Cell lysates were analysed for the levels of proteins of interest with their relevant antibodies.

### Western blotting

Vero cells were infected with PEDV HXLV strain (0.5 MOI) and then incubated for 0, 4, 8, and 16 hr or infected with various MOI (0, 0.005, 0.05, 0.5) for 16 hr. IPEC-DQ cells infected with PEDV HLJBY strain (0.5 MOI) and incubated for 0, 12, 24, and 36 hr or infected with various MOI (0, 0.005, 0.05, 0.5) and then incubated for 16 hr (Figure 1(a,b)). The concentrations of various inhibitors and their incubation time after addition into cell culture were given in figure legends. The levels of interested proteins and their phosphorylation were analysed by Western blot as previous reported [51].

### Cell viability assay

The confluent monolayer of Vero cells seeded in 96-well plates was mock-infected or infected with PEDV (0.5 MOI) and then incubated for 12 hr without or with the addition of indicated inhibitors. Cell viability was measured by using an ATP-based CellTiter-Glo kit (Promega, Madison, WI, USA) according to the manufacturer’s instruction.

### Statistical analysis

An unpaired Student *t* test was used to assess the statistical differences in virus titres, cell viability, the number of autophagosomes and autolysosomes, and the Western blot band density. A *p* value of <0.05 was regarded as being statistically significant. SigmaPlot 11 software (Systat Software, Inc, San Jose, CA, USA) was used to calculate all *p* values.

## Supplementary Material

Supplemental Material

## Data Availability

The authors confirm that the data reported in this study are available within the article and/or its supplementary materials.
