# Supplementary figures and images for: Porcine epidemic diarrhoea virus (PEDV) infection activates AMPK and JNK through TAK1 to induce autophagy and enhance virus replication
Source: Virulence. 2022 Sep 27;13(1):1697–712. doi: 10.1080/21505594.2022.2127192 (PMC9543055; doi:10.1080/21505594.2022.2127192)

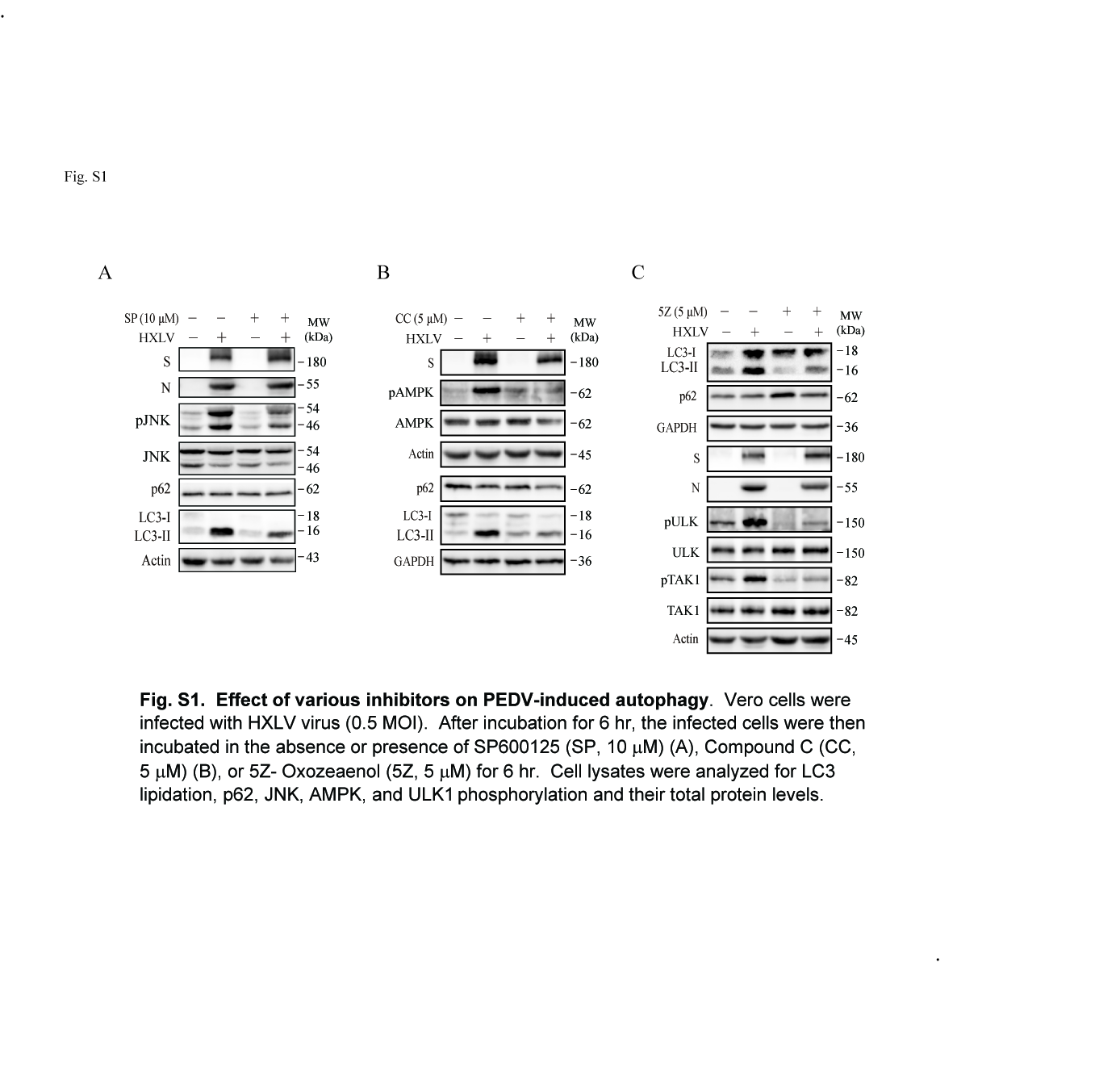

Supplement: Supplemental Material [file KVIR_A_2127192_SM5444.tif]
